# A fundus vessel segmentation method based on double skip connections combined with deep supervision

**DOI:** 10.3389/fcell.2024.1477819

**Published:** 2024-10-03

**Authors:** Qingyou Liu, Fen Zhou, Jianxin Shen, Jianguo Xu, Cheng Wan, Xiangzhong Xu, Zhipeng Yan, Jin Yao

**Affiliations:** ^1^ College of Mechanical and Electrical Engineering, Nanjing University of Aeronautics and Astronautics, Nanjing, China; ^2^ The Affiliated Eye Hospital of Nanjing Medical University, Nanjing, China; ^3^ College of Electronic and Information Engineering, Nanjing University of Aeronautics and Astronautics, Nanjing, China

**Keywords:** fundus vessel segmentation, double skip connections, deep supervision, transformer, vessel feature extraction

## Abstract

**Background:**

Fundus vessel segmentation is vital for diagnosing ophthalmic diseases like central serous chorioretinopathy (CSC), diabetic retinopathy, and glaucoma. Accurate segmentation provides crucial vessel morphology details, aiding the early detection and intervention of ophthalmic diseases. However, current algorithms struggle with fine vessel segmentation and maintaining sensitivity in complex regions. Challenges also stem from imaging variability and poor generalization across multimodal datasets, highlighting the need for more advanced algorithms in clinical practice.

**Methods:**

This paper aims to explore a new vessel segmentation method to alleviate the above problems. We propose a fundus vessel segmentation model based on a combination of double skip connections, deep supervision, and TransUNet, namely DS2TUNet. Initially, the original fundus images are improved through grayscale conversion, normalization, histogram equalization, gamma correction, and other preprocessing techniques. Subsequently, by utilizing the U-Net architecture, the preprocessed fundus images are segmented to obtain the final vessel information. Specifically, the encoder firstly incorporates the ResNetV1 downsampling, dilated convolution downsampling, and Transformer to capture both local and global features, which upgrades its vessel feature extraction ability. Then, the decoder introduces the double skip connections to facilitate upsampling and refine segmentation outcomes. Finally, the deep supervision module introduces multiple upsampling vessel features from the decoder into the loss function, so that the model can learn vessel feature representations more effectively and alleviate gradient vanishing during the training phase.

**Results:**

Extensive experiments on publicly available multimodal fundus datasets such as DRIVE, CHASE_DB1, and ROSE-1 demonstrate that the DS2TUNet model attains F1-scores of 0.8195, 0.8362, and 0.8425, with Accuracy of 0.9664, 0.9741, and 0.9557, Sensitivity of 0.8071, 0.8101, and 0.8586, and Specificity of 0.9823, 0.9869, and 0.9713, respectively. Additionally, the model also exhibits excellent test performance on the clinical fundus dataset CSC, with F1-score of 0.7757, Accuracy of 0.9688, Sensitivity of 0.8141, and Specificity of 0.9801 based on the weight trained on the CHASE_DB1 dataset. These results comprehensively validate that the proposed method obtains good performance in fundus vessel segmentation, thereby aiding clinicians in the further diagnosis and treatment of fundus diseases in terms of effectiveness and feasibility.

## 1 Introduction

**FIGURE 1:**
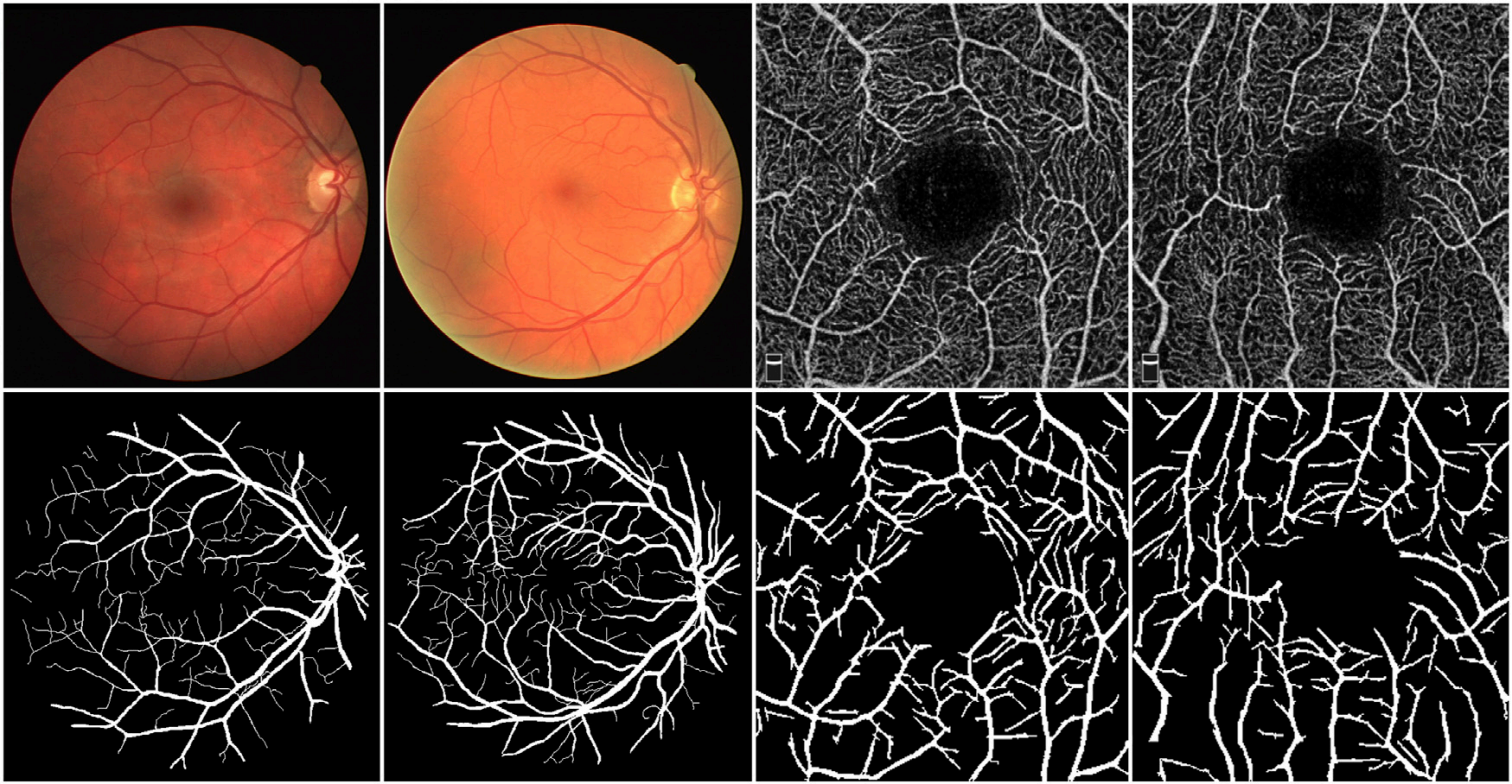
Upper row: CF images and OCTA images. Bottom row: Corresponding label images.

The retina is the sole part of the human organism where arteries, veins, and capillaries are visible to the naked eye. Fundus images contain richer contextual structural information compared to most natural images, offering critical clinical insights for physicians. Analyzing the shape, bifurcation, and other structural features of blood vessels in color fundus (CF) images enables physicians to diagnose diseases such as diabetic retinopathy, microaneurysms, and hypertension (Fraz et al., 2012). Furthermore, research has demonstrated that the vessel structure in optical coherence tomography angiography (OCTA) images is specifically and distinctly altered in patients with Alzheimer’s disease (AD) and those with mild cognitive impairment (MCI) (Chua et al., 2020). Hence, investigating morphological changes in blood vessels offers substantial diagnostic value, particularly for observing and detecting deeper branch vessels and microvessel details. To fully leverage this diagnostic potential, accurate segmentation of fundus vessel information is crucial, as it serves as a vital indicator for diagnosing ocular diseases. As illustrated in Figure 1, the top row presents the raw data of CF and OCTA images, while the bottom row depicts their corresponding vessel segmentations.

However, segmenting fundus blood vessels faces substantial challenges due to the complex structure of the vessel network in fundus images, uneven gray scale distribution, low contrast between blood vessels and background, and the influence of image noise. Researchers have proposed a series of schemes for fundus vessel segmentation. These schemes use both traditional and deep learning approaches.

### 1.1 Traditional segmentation methods

Traditional segmentation methods rely on manual feature selection, followed by vessel segmentation using appropriate classifiers. Chaudhuri et al. (1989) initially suggested the segmentation of fundus images using a Gaussian filter, which encoded blood vessel features and matched these encoded features using a two-dimensional Gaussian filter. This method addressed the issue of low contrast between blood vessels and the background. Azzopardi et al. (2015) introduced the B-COSFIRE (Blood-Combination of Shifted Filter Responses) filter, which selectively responded to blood vessels for automatic vessel segmentation. This was achieved by calculating the weighted average of the Gaussian filter outputs for directional selection and ensuring translation without distortion through a simple shift operation. The authors employed two B-COSFIRE filters, symmetric and asymmetric, which segmented blood vessels by summing the responses of the two filters and setting a threshold. Li et al. (2012) developed a segmentation method that combined multi-scale matched filtering and dual thresholding. This method strengthened vessel models by multiplying the responses from three scales of matched filtering, resulting in effective vessel segmentation.

### 1.2 CNN-based segmentation methods

Compared to traditional segmentation algorithms, pixel-level semantic segmentation algorithms based on deep learning can fully extract semantic feature information. This allows them to make accurate predictions for each pixel category, resulting in higher precision and accuracy. Shelhamer et al. (2017) pioneered the Fully Convolutional Network (FCN). They adapted the classification network to a fully convolutional form, which marked the first successful application of deep learning to image semantic segmentation. This surpassed the state-of-the-art technology in the field of semantic segmentation at that time. Ronneberger et al. (2015) designed U-Net based on FCN to better fuse image features. This design achieved faster processing speeds, required fewer training images, and demonstrated greater suitability for medical segmentation tasks, such as retinal blood vessels. However, there is still room for improvement as feature information can be easily lost during the down-sampling process, resulting in inadequate blood vessel segmentation accuracy. In response, several better algorithms based on U-Net have been developed. Wu et al. (2019) created a new lightweight vessel segmentation model, Vessel-Net, for fundus images. This model incorporated an efficient initial residual convolution block into the U-Net structure. By merging the benefits of U-Net and residual modules, it boosted feature representation and significantly improved segmentation performance. Jin et al. (2019a) introduced DU-Net (Deformable U-Net) for vessel segmentation of fundus images. They applied deformable convolution to the U-Net architecture. This approach adaptively adjusted the receptive field according to differences in vessel morphology and scale. It used an up-sampling operator to increase the resolution of the output image by combining encoder and decoder features. This combination allowed for the extraction of contextual information and precise localization, resulting in accurate segmentation of retinal blood vessels. Mou et al. (2021) suggested adding an attention module to each encoder and decoder of U-Net. These two attention modules were used to enhance inter-class discrimination and intra-class response, respectively. They also raised the use of 1 × 3 and 3 × 1 convolution kernels to capture boundary features. Experimental results on multiple datasets verified the effectiveness of this method. Kwon (2021) put forward an advanced adversarial training method to resist the interference of unknown adversarial examples. The authors used the Fast Gradient Sign Method (FGSM) to generate a fixed range of adversarial examples for training. Experiments established that U-Net models trained on different adversarial examples yielded better segmentation results on unknown adversarial examples. Fu et al. (2016) introduced DeepVessel, which integrated deep learning with Conditional Random Fields (CRFs) to enhance retinal vessel segmentation by capturing fine structures, though this approach added to the model’s computational demands. Alom et al. (2019) expanded on traditional U-Net models with the Recurrent Residual U-Net, incorporating recurrent layers and residual connections to improve feature refinement, allowing for deeper network training and better segmentation performance. Li et al. (2020) introduced MAU-Net, which utilized multi-scale feature extraction and attention mechanisms to improve segmentation accuracy, particularly for vessels of varying sizes. Gu et al. (2019) presented CE-Net, which enhanced boundary accuracy by integrating context encoding within an encoder-decoder framework, making it particularly effective in refining segmentation results and improving the delineation of retinal vessels. Jin et al. (2019b) developed DUNet, leveraging deformable convolutions that adapted to the shape and size of retinal vessels, leading to more accurate segmentation of complex vessel morphologies. Mou et al. (2019) created CS-Net, which used channel and spatial attention mechanisms to enhance vessel segmentation, effectively capturing fine details and improving overall accuracy. Guo (2023) introduced the DPN (Detail-Preserving Network) for retinal vessel segmentation, avoiding traditional encoder-decoder architectures like U-Net by maintaining high-resolution feature maps with Detail-Preserving Blocks. This design significantly enhances both segmentation accuracy and speed, especially on the DRIVE dataset. Alvarado-Carrillo and Dalmau-Cedeño (2022) proposed a Width Attention-based CNN that focuses on vessel thickness during segmentation, excelling in capturing thin and irregular vessels but potentially facing challenges with complex vessel networks. Liu et al. (2023) developed ResDO-UNet, incorporating deep residual connections into the U-Net architecture to improve gradient flow and feature learning, resulting in higher segmentation accuracy, albeit with increased computational costs. Kumar et al. (2023) introduced IterMiUnet, a lightweight CNN designed for blood vessel segmentation. They adopted an iterative mechanism to refine results, making it suitable for resource-constrained environments. However, it may have slower inference times. These methods exhibited advancements in retinal vessel segmentation by tackling key challenges such as feature refinement, multi-scale processing, and attention mechanisms.

### 1.3 CNN-transformer segmentation methods

Various Transformer (Vaswani et al., 2017) architectures, known for their ability to capture global attention, were used to compensate for the limitations of convolutional inductive bias (Cao et al., 2023). However, due to the quadratic complexity of self-attention and the lack of convolution-like inductive bias, models based on the original Transformer could only achieve optimal results on large-scale datasets (Dosovitskiy et al., 2021). In addition to the limitations of dataset size, although some studies (Wang et al., 2022; Zheng et al., 2021) showed that Transformer could outperform CNN and had the potential to fully replace convolutional blocks, it did not consistently outperform CNN in all tasks. Zhang et al. (2023) pointed out that segmentation tasks required not only improved global context modeling but also a focus on low-level details. Therefore, segmentation networks based solely on Transformer tended to yield suboptimal results. Chen et al. (2021) introduced TransUNet (Transformer and U-Net) for medical image segmentation, applying the Transformer model originally developed for natural language processing. It combined the advantages of the Transformer and U-Net and achieved favorable results in various medical applications such as multi-organ segmentation and heart segmentation. Yang and Tian (2022) extended TransUNet with TransNUNet, enhancing the upsampling stage with a convolutional attention module. This modification allowed the model to focus more effectively on relevant features while suppressing irrelevant ones, improving segmentation accuracy by selectively attending to critical areas of the image. And Haonan et al. (2022) rethought U-Net’s skip connections by incorporating a channel Transformer to connect multi-scale channel information to the decoder, although spatial and boundary information challenges remained. Wang et al. (2023) developed a cross-convolution Transformer for medical image segmentation. The novel cross-convolution self-attention mechanism integrated local and global context, modeling both long-range and short-range dependencies to enhance the understanding of semantic features. This study also proposed a multi-scale feature edge fusion module to fuse edge features of images, improving the problem of blurred target edge contours. In 2023, Tragakis et al. (2023) introduced the full-convolution Transformer, the first fully convolutional Transformer architecture in the field of medical image segmentation. This model effectively learned long-range semantic information through an innovative convolutional attention module and created a hierarchical local-to-global context structure using the fully convolutional Wide-Focus module. Global and local information were crucial for image segmentation tasks. By combining CNN with Transformer, the strengths of both architectures were fully utilized. Specifically, convolution operations captured detailed features in local regions, while the Transformer structure provided overall context and structural information. This fusion of local features and global context improved segmentation performance.

Nevertheless, the limitations of traditional segmentation methods and deep learning segmentation algorithms in dealing with the task of accurately segmenting vessel structures cannot be overlooked. From a technical point of view, traditional segmentation methods tend to rely on expert experience and specific parameter settings to extract features when segmenting blood vessel information. These methods are more suitable for data with high image quality and clear vessel structure. They struggle with scenes that have minimal contrast variation and are unable to fully access the rich vessel information contained in fundus images. Deep learning algorithms are powerful in capturing vessel features, but the current shortage of medical image data inevitably leads to issues of model overfitting and insufficient generalization. Moreover, from an application perspective, the aforementioned methods are predominantly applied to unimodal images. One method may excel in handling vessel segmentation tasks in the CF images, yet its vessel representation ability may not be effectively extended to the OCTA images.

Therefore, based on the above analysis, to address the issues of insufficient segmentation of fundus vessel details and weak generalization of existing algorithms, we put forward a novel vessel segmentation model called DS2TUNet based on the TransUNet, to achieve accurate segmentation of vessel structures and boost the generalization performance of the vessel segmentation model. This motivation drives the following contributions of this paper:

(1) The dual downsampling operation generates two sets of sampled features for the double skip connection module. ResNetV1 downsampling focuses on local feature extraction using residual connections, while dilated convolution downsampling captures global information by expanding the receptive field. The Transformer integrates both local and global features, enhancing vessel extraction.
(2) The double skip connection module combines these intermediate features and passes the concatenated map to the decoder’s upsampling layer. This enriches feature fusion, retains detailed information, and improves segmentation accuracy compared to layer-by-layer passing alone.
(3) The deep supervision module supervises training through segmentation results from different layers. It mitigates gradient vanishing and enhances segmentation accuracy, particularly in complex vessel structures.

The remaining structure of this paper is as follows: Section 2 describes related works. Section 3 details the implementation of our proposed method. Section 4 presents the materials and the experiments and discussions. Section 5 concludes the research.

## 2 Related work

### 2.1 TransUNet

TransUNet employs the R50-ViT-B_16 model as its foundation to enhance feature extraction and segmentation accuracy. This module serves as a feature extractor, comprising ResNet50 (Carion et al., 2020) integrated with the ViT (Dosovitskiy et al., 2021) (Vision Transformer). ResNet50 excels at extracting rich low-level features from an image, capturing fine structure and texture details that enhance its feature extraction capabilities. When an image is input, high-level feature extraction is initially performed using the ViT model to obtain global features. These high-level features are then combined with the low-level features extracted by ResNet50. The intermediate and low-level feature maps obtained by the feature extractor are connected to the decoding module’s feature maps through skip connections. This design leverages ResNet50’s strengths in low-level feature extraction and ViT’s proficiency in capturing high-level global features. By effectively fusing features at different levels, it significantly enhances the performance and accuracy of TransUNet.

The input image dimensions are transformed into a series of sequences with positional information through a patch embedding module. This transformation allows for parallel processing and efficient utilization of global information. The input image $\left.\left\{x\in \right.R^{H\times W\times C}\right\}$ is tokenized and transformed into a series of 2D patch sequences $\left\{x_{p}^{i}\in R^{P^{2}C}\left|\left.i=1,\ldots \ldots ,N\right\}\right.\right.$ . Where H×W is the image resolution, C is the number of image channels, $P^{2}$ is the size of each patch, and $N=\frac{HW}{P^{2}}$ is the number of patches.

**FIGURE 2:**
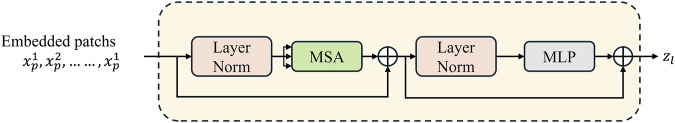
Transformer layer structure (Chen et al. 2021).

The Transformer module contains 12 layers of the Transformer network, and each encoder layer consists of a multi-head self-attention mechanism (MSA), a multi-layer-perceptron (MLP), and a layer normalization (LN). The sequence outputs after MSA and MLP are represented by Equations 1 and 2. The structure of the Transformer layer is illustrated in Figure 2.

$$z_{l}^{\prime }=MSA\left(\mathrm{LN}\left(z_{l-1}\right)\right)+z_{l-1}$$ (1)

$$z_{l}=MLP\left(\mathrm{LN}\left(z_{l}^{\prime }\right)\right)+z_{l}^{\prime }$$ (2)

where $z_{l-1}$ is the output of the previous layer, $z_{l}$ is the output of layer l and serves as the input to the next layer, and $z_{l}^{\prime }$ is the output of the MSA connected to the residual of $z_{l-1}$ .

**FIGURE 3:**
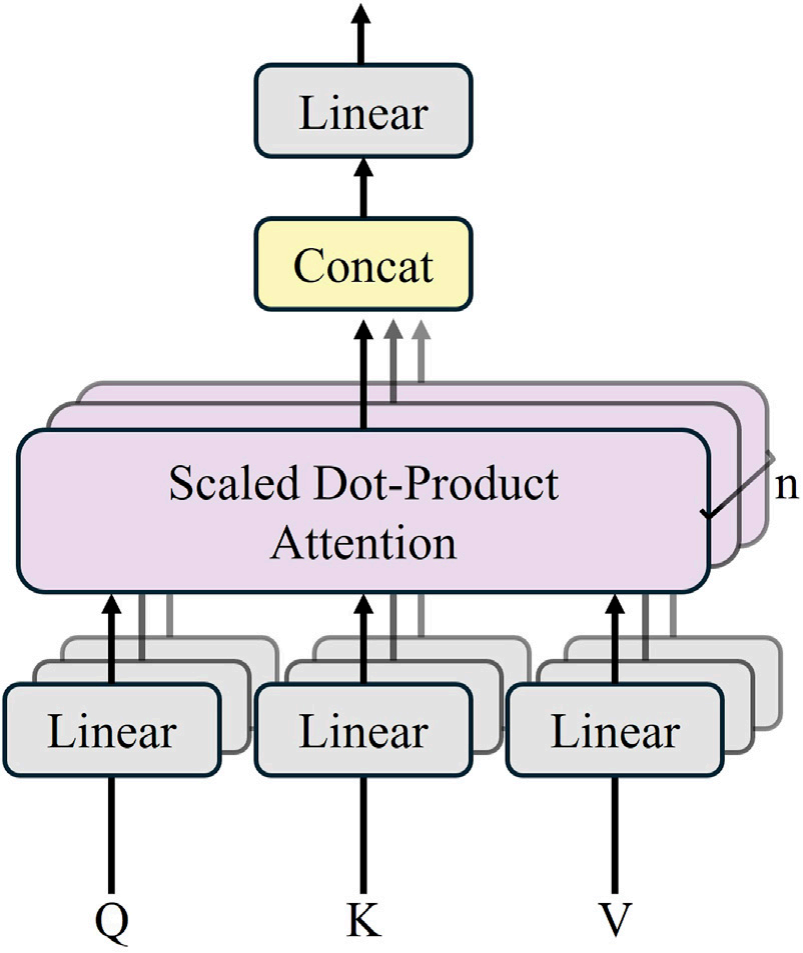
MSA structure.

TransUNet employs a hybrid CNN-Transformer architecture as an encoder to achieve precise localization (Carion et al., 2020). The structure is shown in Figure 3.

In the multi-head self-attention mechanism, multiple q , k , and v vectors form matrix Q , K , and V , respectively. The parameters of each combination are decomposed into different subspaces to compute the attention weights. After several parallel computations, the results are concatenated in the channel dimension and combined to obtain the attention information in all subspaces. Where Q is the query matrix, K is the key matrix, and V is the value matrix.

TransUNet introduces the cascaded upsampler (CUP), consisting of multiple upsampling modules. Each module is tasked with upsampling the feature map and fusing it with the feature map from the subsequent level. This cascaded structure facilitates a gradual increase in resolution and the fusion of information across multiple scales. The feature sequence output from the Transformer is reshaped by implementing the CUP with four upsampling blocks. The reshaped feature maps are subsequently upsampled three times in sequence to produce feature maps with varying dimensions. The skip connection is employed to reach the fusion of features at different resolution levels and to recover the low and intermediate-level details lost during downsampling.

TransUNet allows each feature decoder to employ bilinear interpolation as an upsampling method, which increases the resolution of the feature maps to match the original input image dimensions, thereby recovering the segmentation results. Furthermore, the outputs of the feature encoder and feature decoder are connected via skip connection to retain more details, thereby augmenting segmentation accuracy.

### 2.2 ResNetV1

ResNet (Residual Network) is a widely adopted deep convolutional neural network architecture, initially designed by He et al. (2016). The version of ResNet implemented in TransUNet is ResNetV1. The core concept of ResNetV1 is the introduction of “shortcut connections,” which allows the network to directly learn the residuals between inputs and outputs. A residual block typically comprises two or three convolutional layers, each immediately followed by the Batch Normalization and a ReLU activation function. The shortcut connections can be either identity mappings, where the input is directly added to the output, or dimension-matching connections using 1 × 1 convolutional layers. The fundamental structure of ResNetV1 consists of multiple residual blocks stacked on top of each other. It is available in several variants, including ResNet-18, ResNet-34, ResNet-50, ResNet-101, and ResNet-152. For instance, ResNet-50 begins with a convolutional and pooling layer (Conv + MaxPool), followed by four stages, each stage containing a different number of residual blocks, and concludes with a global average pooling layer and a fully connected layer. ResNetV1 is extensively utilized in tasks such as image classification, object detection, and semantic segmentation. Its simple and efficient architecture is straightforward to implement across various deep learning frameworks.

## 3 The proposed methods

### 3.1 Structure of the improved model

**FIGURE 4:**
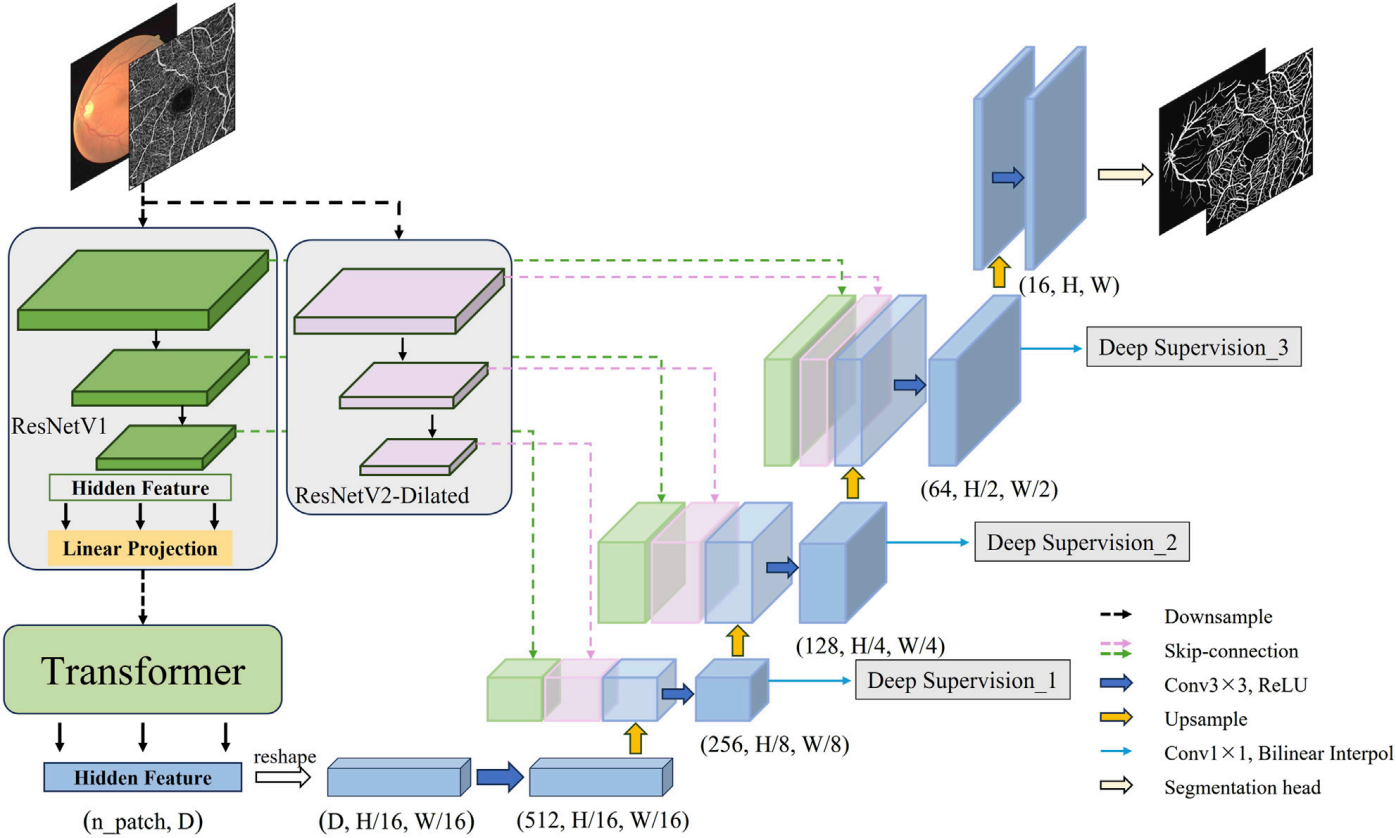
The DS2TUNet model structure.

On basis of the aforementioned technical research and inspiration from the TransUnet architecture, this section officially constructs a novel fundus vessel segmentation model. The structure of the improved model is illustrated in Figure 4. First, through the dual downsampling operation of ResNetV1 downsampling and dilated convolution downsampling, two sets of feature maps with different scales and receptive fields are extracted from the fundus images. This allows for fully capturing the contextual information of the complex fundus vessel structures, achieving better feature representation. The final output hidden features from ResNetV1 downsampling are then serialized and fed into the Transformer encoder for processing and reassembling into a complete feature map. The downsampling features are then fused by double skip connections at the upsampling stage. Finally, a deep supervision signal is added to each upsampling output feature and weighted to compute the target loss. The high-resolution feature maps are input to the segmentation header to obtain the final segmentation result.

### 3.2 Methods

The construction of DS2TUNet is primarily introduced in three parts: the dual downsampling operation, the double skip connection module and the deep supervision module. The model improves fundus image feature extraction by integrating ResNetV2. The downsampled intermediate features are fed into the model’s upsampling stage through the double skip connection module. This process effectively integrates both global and local features. Finally, the utilization of intermediate output features of the decoder is further optimized through deep supervision, which strengthens segmentation accuracy.

### 3.3 Dual downsampling operation

**FIGURE 5:**
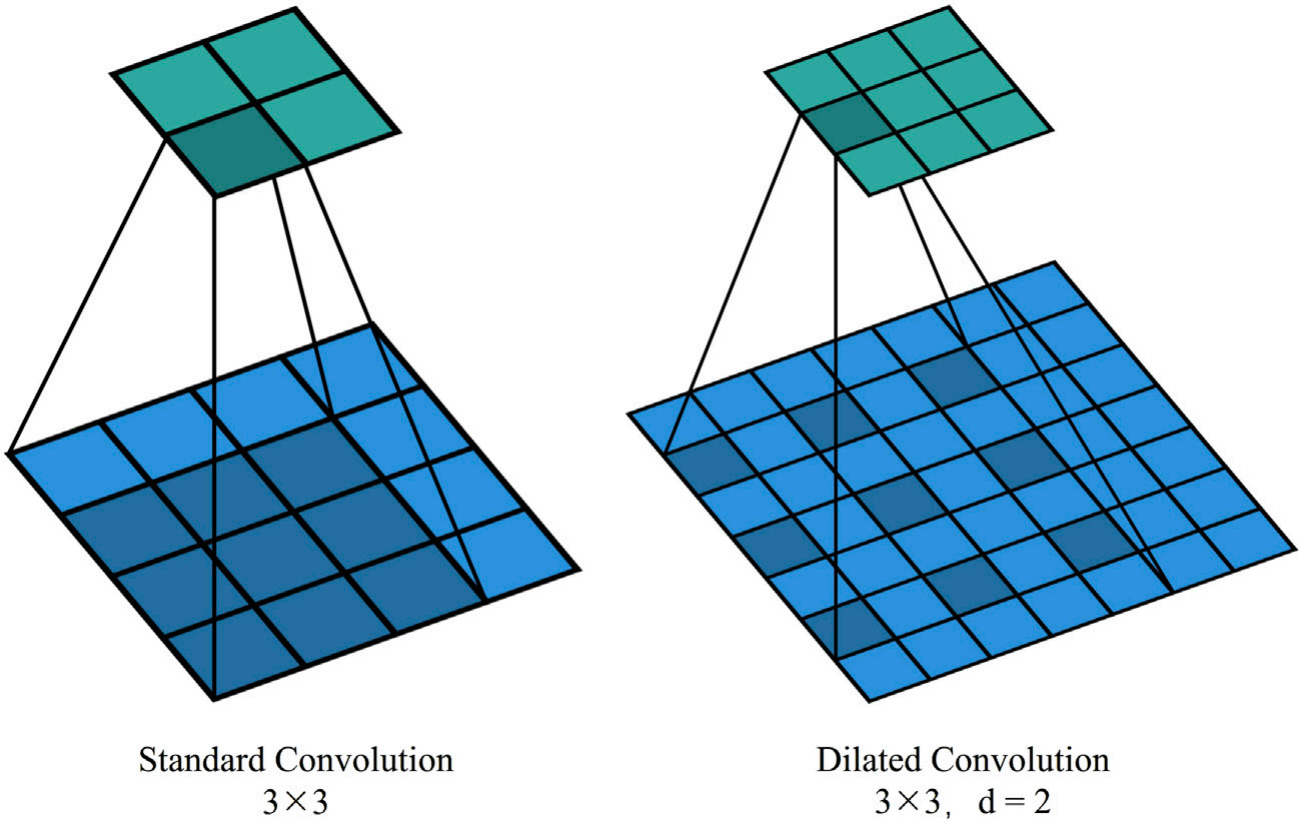
Different convolutional structures.

In TransUNet’s original downsampling structure, convolution and pooling operations have a limited receptive field, often resulting in a loss of contextual feature information. Introducing dilated convolution, which inserts cavities between elements in the convolution kernel, expands the receptive field without increasing the number of parameters and computational complexity. By replacing traditional standard convolutional operations, it becomes possible to capture a broader range of contextual information. This boost is particularly beneficial for handling complex physiological structures like the fundus vasculature, which exhibits long-distance dependencies. This is illustrated in Figure 5, which shows standard convolution and dilated convolution with a dilation rate of 2.

In our model, the original downsampling structure is retained, and a dilated convolution downsampling structure is added in parallel to obtain a broader range of contextual information for feature enhancement. The added downsampling module is further optimized using the ResNetV2 structure. The overall structure of ResNetV2 is similar to ResNetV1, but the order within each residual block is different. In ResNetV2, the positions of the Batch Normalization (BN) and ReLU activation functions are moved to before the convolutional layer from after it.

Residual operations are described in Equations 3 and 4

$$y_{ResNetV1}=x+\text{ReLU}\left(\text{BN}\left(\text{Conv}\left(x\right)\right)\right)$$ (3)

$$y_{ResNetV2}=x+\text{Conv}\left(\text{ReLU}\left(\text{BN}\left(x\right)\right)\right)$$ (4)

**FIGURE 6:**
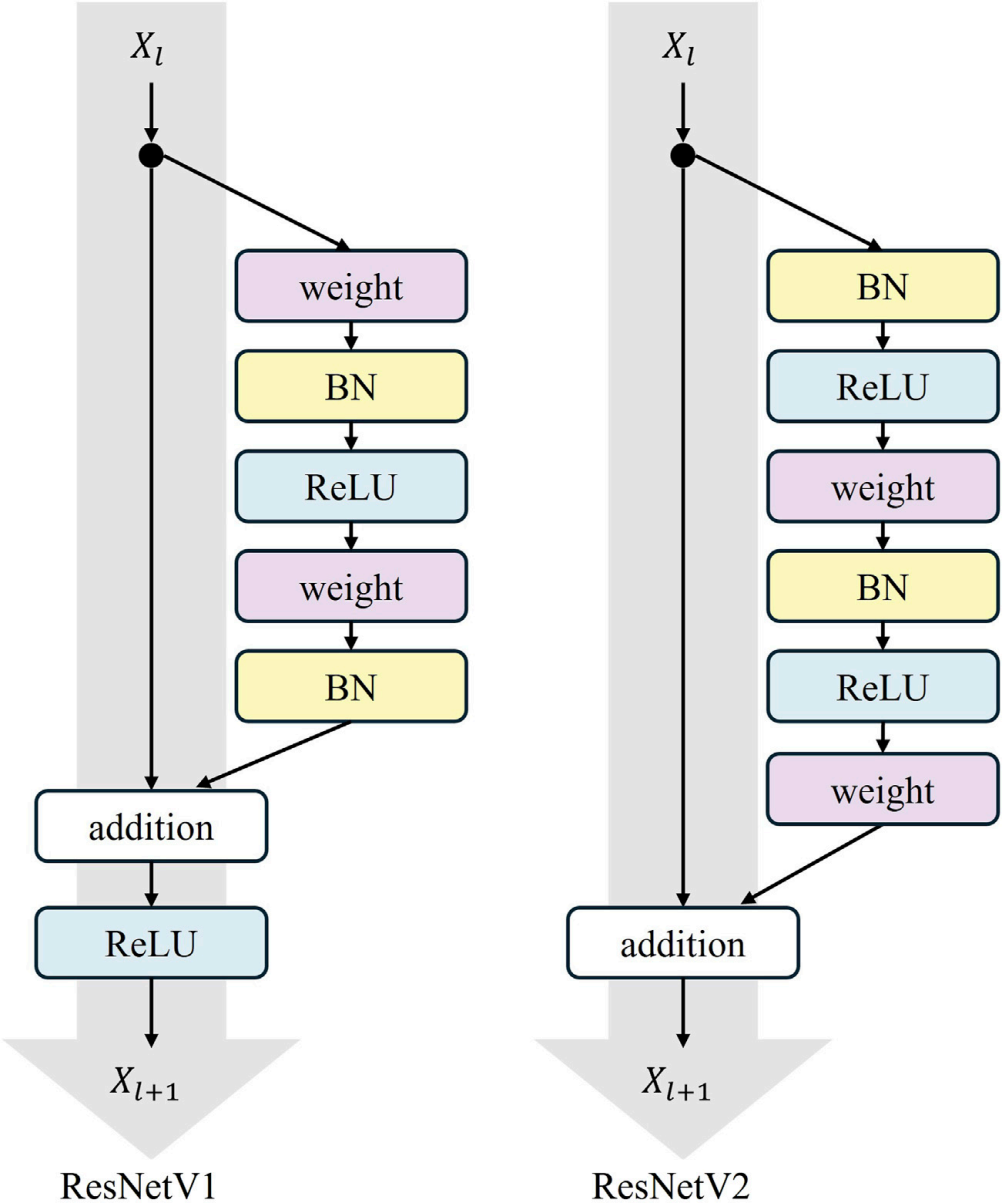
Residual block structures of ResNet.

This augmentation is termed the “Pre-activation Residual Block,” which helps facilitate gradient flow and mitigates the gradient vanishing problem. The pre-activation design provides regularization by normalizing the inputs to the convolutional layers. This normalization reduces the internal covariate shift, which is the change in the distribution of layer inputs during training. This reduction achieves a more robust and generalized model that performs better on unseen data. The specific structure of the residual block is shown in Figure 6, where “weight” refers to the convolutional layer.

The architectural advancements in ResNetV2 enhance performance and training stability, especially for deep networks. This makes ResNetV2 a compelling choice for a variety of applications. The improved dual downsampling operation augments the extraction of global and local features of the fundus vasculature and provides input data for subsequent double skip connections for feature fusion.

### 3.4 Double skip connection module

Skip connection is a feature of the traditional U-Net, where feature maps from the encoder are passed directly to the corresponding level of feature maps in the decoder. This connection helps preserve low-level feature information and avoid information loss, which enhances the recovery of microvessel details in fundus images and improves segmentation accuracy. Additionally, skip connections allow gradients to pass directly, thus improving gradient flow and mitigating the gradient vanishing problem.

**FIGURE 7:**
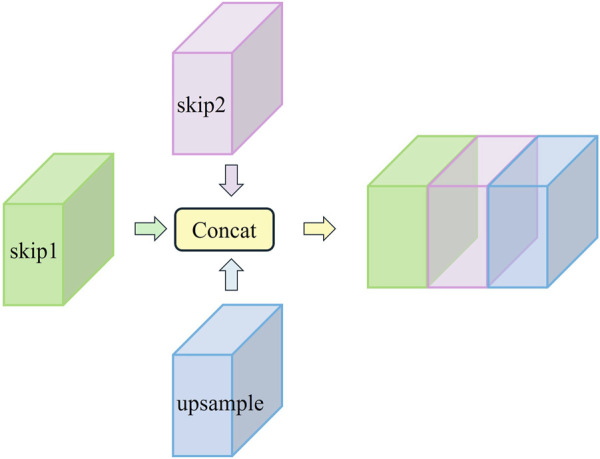
Double skip connection module structure.

However, the relatively limited receptive field of this operation still results in the loss of some contextual information. To address this issue mentioned in the previous subsection, we generate additional feature information using dilated convolution while preserving the original downsampling features. After downsampling each layer, we obtain both the original features and the dilated convolution features. These features are passed to the corresponding layer through two skip routes and then concatenated with features from the upsampling process along the channel dimension. This process forms the double skip connection module. The specific structure of the double skip connection module is shown in Figure 7.

This approach not only helps retain detailed information but also addresses the gradient vanishing issue often encountered in deep architectures. Specifically, this module utilizes two skip routes. Skip1 passes the original downsampling features from the traditional U-Net, helping to preserve low-level feature information and retain microvessel details during the decoding process. Skip2 delivers features generated by dilated convolution, which expands the receptive field and captures more contextual information, aiding in the accurate identification of microvessels. These two sets of features are then concatenated along the channel dimension with the upsampling features, creating a rich feature fusion.

Assuming the original feature map obtained at layer l in the encoder is $X_{l}$ , the feature map obtained by dilated convolution is $D_{l}$ , and the feature map obtained at layer L−l in the decoder is $Y_{L-l}$ , then the skip connection can be represented by Equation 5.

$$Y_{L-l}=Upsample\left(Y_{L-\left(l+1\right)}\right)+X_{l}+D_{l}$$ (5)

One of the key advantages of the double skip connection module is its impact on gradient flow. By providing dual pathways for feature propagation, the module mitigates the vanishing gradient problem more effectively than single skip connection. The dual pathways allow for a smoother gradient flow, especially in deep networks, thereby enhancing the stability and convergence speed of the training process. The visual representation of this module in Figure 7 clearly illustrates the flow of features through the double skip connection. The original features (skip1) and dilated features (skip2) are merged with the upsampling features to form a comprehensive feature map that is rich in both local and global information. It excels in preserving micro-level details while capturing macro-level structures. This is critical for tasks like fundus vessel segmentation, where both small capillaries and larger vessels must be accurately segmented.

To validate the impact of the double skip connection module, ablation studies were conducted on fundus image datasets. These studies compared the performance of the DS2TUNet model with and without the double skip connections. The results showed a marked improvement in segmentation performance when the double skip connections were employed, which underscores the module’s contribution to the model’s overall performance.

Introducing double skip connections further utilizes global contextual information to enable the model to better understand and process complex structures in images, particularly fundus vessel structures with complex topologies and long-distance dependencies. This allows the model to better adapt to different types of images and structures, thereby enhancing its generalization ability across various datasets.

### 3.5 Deep supervision module

The model utilizes an end-to-end network structure to calculate the loss by introducing deep supervision. The target loss function consists of two components: the deep supervision auxiliary loss ( $L_{DS}$ ) and the main segmentation loss ( $L_{MS}$ ). The model incorporates deep supervision signals at each layer of the upsampling output results. It applies convolution operations to adjust the channel number of the upsampling output and uses bilinear interpolation to adjust its size. The loss is then computed with the ground truth at different layers.

The model’s basic loss function is computed using cross-entropy loss:

$$L_{CE}=-\frac{1}{N}\sum_{i=1}^{N}\left(y_{i}\log\left({\hat{y}}_{i}\right)+\left(1-y_{i}\right)\log\;\left(1-{\hat{y}}_{i}\right)\right)$$ (6)

where N is the number of pixels in the input image, ${\hat{y}}_{i}$ is the predicted output probability of the pixel, and $y_{i}$ is the true value pixel. $L_{MS}$ is the loss value computed using the cross-entropy loss function for the main segmentation result.

The $L_{DS}$ is computed from the loss function:

$$L_{DS}=\sum_{i=1}^{3}\frac{1}{2^{i}}{L_{CE}}_{i}$$ (7)

where the hyperparameter $\frac{1}{2^{i}}$ is the weight coefficient of the deep supervision auxiliary loss. Since the deeper the layer in upsampling, the greater the risk of gradient vanishing, larger weights are assigned to the deeper layers in the upsampling. Combining Equations 6 and 7, the final objective function is formulated as shown in Equation 8.

$$L=L_{DS}+L_{MS}$$ (8)

## 4 Results and discussions

### 4.1 Fundus image datasets and experimental settings

The fundus image datasets utilized in the experiment comprises two types: the public CF datasets, including DRIVE and CHASDE_DB1, and the public OCTA dataset ROSE-1, as well as a clinical dataset provided by the Affiliated Eye Hospital of Nanjing Medical University with patients diagnosed with Central Serous Chorioretinopathy (CSC). Patients’ privacy information has been meticulously desensitized. All images have corresponding labeled images. The public datasets are divided into training and testing sets, while the clinical dataset is exclusively designated for testing to further validate the model’s effectiveness. The DRIVE dataset consists of 40 pairs, with 20 pairs in the training set and 20 pairs in the testing set. The CHASE_DB1 dataset contains 28 pairs, with 200 pairs in the training set after data augmentation and 8 pairs in the testing set (Su et al., 2023). The ROSE-1 dataset includes 30 pairs, with 200 pairs in the training set after data augmentation and 10 pairs in the testing set. The CSC dataset consists of 20 pairs, all of which are allocated for testing.

Table 1 presents the epoch, input size, learning rate, batch size, and optimizer settings employed during training for different datasets. Due to the hardware constraints of the training device, the specific settings are tailored to optimize model performance within these limitations.

**TABLE 1:** Parameter settings for different datasets during training phase.

Datasets	Epoch	Input size	Learning rate	Batch size	Optimizer
DRIVE	300	512 × 512	0.005	4	Adam
CHASE_DB1	300	512 × 512	0.005	4	Adam
ROSE-1	300	304 × 304	0.005	8	Adam

The implementation of each module in the proposed method is based on the Python programming language, with the PyTorch framework selected as the development platform for training and testing DS2TUNet. The NVIDIA 4080 graphics card provides the primary computing support.

### 4.2 Evaluation metrics

To evaluate the segmentation performance of the proposed method, we performed qualitative and quantitative analysis. Qualitative analysis involves comparing the loss curves during the training of different models and directly comparing the vessel segmentation results to assess segmentation quality. Quantitative analysis directly compares the values of the following evaluation metrics: Accuracy (ACC), Specificity (SP), Sensitivity (SE) and F1-score. Accuracy indicates the proportion of correctly segmented pixels in the entire image. Specificity indicates the proportion of correctly segmented background pixels. Sensitivity indicates the proportion of correctly segmented blood vessel pixels. F1-score measures the similarity between the segmentation results and the ground truth. The related metrics are calculated as presented in Equations 9–12.

$$ACC=\frac{TP+TN}{TP+TN+FP+FN}$$ (9)

$$SP=\frac{TN}{TN+FP}$$ (10)

$$SE=\frac{TP}{TP+FN}$$ (11)

$$F1=\frac{2TP}{2TP+FP+FN}$$ (12)

where TP (True Positive) represents pixels correctly predicted as vessel, TN (True Negative) represents pixels correctly predicted as non-vessel, FP (False Positive) represents pixels incorrectly predicted as vessel, and FN (False Negative) represents pixels incorrectly predicted as non-vessel.

### 4.3 Discussion on the dilation rate of the double skip connection module

Dilated convolution extends the receptive field without adding additional parameters or increasing computational complexity. This helps in capturing both global and detailed information, making it suitable for segmenting complex structures like fundus blood vessels. However, it can suffer from the gridding effect, which may lead to sparse feature maps and information loss, affecting the capture of fine structures. Therefore, a suitable dilation rate needs to be carefully selected to balance the extension of the receptive field with the effectiveness of feature extraction. By comparing the results of the base model and models with dilation rates of 2, 3, 5, and 7, the impact of dilated convolutions on overall performance can be understood. The dilation rates of 2, 3, 5, and 7 are selected for the comparison experiments because a dilation rate of 1 is practically equivalent to a standard convolution. These rates include small, medium, and large dilations. They enable a comprehensive assessment of how dilated convolutions impact model performance. Before the experimental analysis, the meanings of relevant terms involved in the process are explained in advance. BM means base model, and DR is an abbreviation for dilation rate, and the number immediately following it represents the size of the dilation rate, e.g. DR-2 means a dilution rate of 2. The fundus images used for the experiment is from the DRIVE dataset.

#### 4.3.1 Qualitative analysis

**FIGURE 8:**
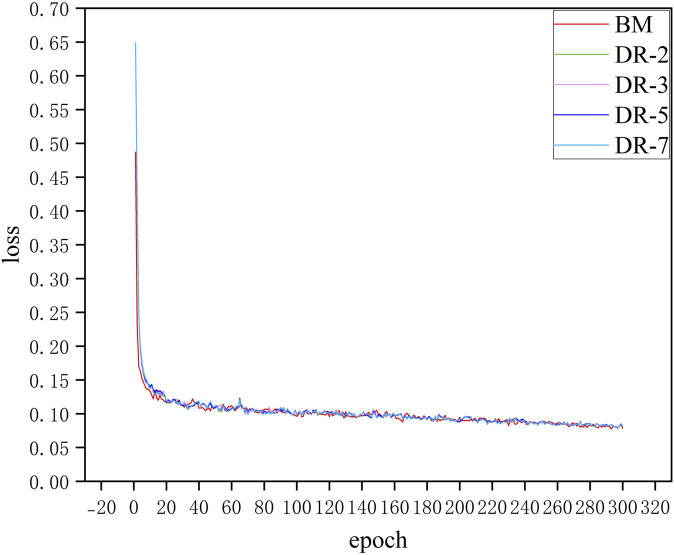
Training loss curves for models with different dilation rates.

Figure 8 shows the loss curves of model with different dilation rates. It can be seen that adding an additional downsampling process does not significantly affect the convergence speed of the model. The loss curves of all models including BM and models with different dilation rates configurations decrease rapidly in the first few epochs, which indicates that the model can effectively learn the characteristics of the data in the initial training phase. Moreover, after about 20 epochs, the loss values of all models tend to be stable and maintain low fluctuations in subsequent training. Although the model loss curves of different dilation rates almost overlap, in practical applications, these small differences may have a significant impact on specific evaluation metrics of the model such as SE and SP. This situation can be demonstrated in the quantitative analysis that follows.

**FIGURE 9:**
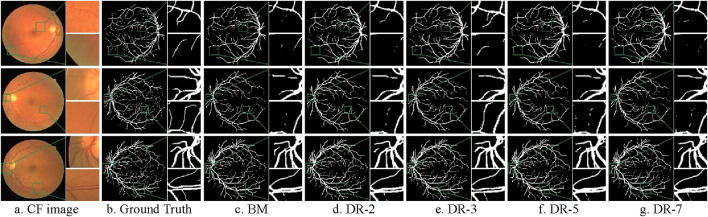
Comparison of model segmentation results with different dilation rates. **(A)** CF image **(B)** Ground Truth **(C)** BM **(D)** DR-2 **(E)** DR-3 **(F)** DR-5 **(G)** DR-7.

Figure 9 illustrates the comparison of the actual segmentation results of the models with different dilation rates. To highlight the differences in segmentation outcomes, we not only provide the segmentation results of the entire fundus images but also zoom in to showcase local details in both typical thick-vessel regions and fine-vessel regions.

It is evident that the base model struggles to accurately capture the continuity of blood vessels, resulting in noticeable gaps and fragmented vessel structures. Additionally, it either fails to detect some blood vessels or over-segments the image, which results in misclassifying background areas as blood vessels. Conversely, models with dilation rates of 3 and 5 demonstrate a significantly improved ability to maintain vessel continuity and accurately delineate blood vessel boundaries. These models reduce instances of both under-segmentation and over-segmentation, providing more precise and reliable segmentation output. Models with dilation rates of 2 and 7, while performing better than the base model, still exhibit some issues. The dilation rate of 2 results in minor blood vessel breakages and occasional over-segmentation, whereas the dilation rate of 7 produces smoother but less precise segmentation, sometimes merging adjacent vessels or missing finer vessel details.

#### 4.3.2 Quantitative analysis

As shown in Table 2, the use of dilated convolution enhances the model’s performance. Bold values in the table indicate the maximum value for this evaluation metric for the models listed. The meaning of the bold values in subsequent tables remains the same. By comparing the base model with models using different dilation rates, substantial advancements in segmentation performance are observed. Considering the evaluation metrics, the dilation rate of 3 emerges as the most suitable configuration for the following reasons:

**TABLE 2:** Comparison of test evaluation metrics for models with different dilation rates.

Model	F1-score	ACC	SE	SP
BM	0.7979	0.9635	0.7701	**0.9845**
DR-2	0.8111	0.9654	0.7945	0.9826
DR-3	0.8117	0.9655	**0.7986**	0.9831
DR-5	0.8115	**0.9656**	0.7960	0.9824
DR-7	**0.8119**	0.9652	0.7960	0.9825

Firstly, the model with a dilation rate of 3 exhibits a substantial amelioration in SE, achieving a value of 0.7986. This represents an increase of approximately 2.85% compared to the base model. This indicates a significant boost in the model’s ability to accurately identify true positives. Additionally, the ACC of the model with a dilation rate of 3 shows a slight improvement, increasing by about 0.2% over the base model.

While the F1-score for the model with a dilation rate of 3 is slightly lower than that of the model with a dilation rate of 7 by about 0.02%, it still shows a notable upgrade of about 1.38% over the base model. Furthermore, the model with a dilation rate of 3 maintains high SP, only around 0.14% below the base model, with a value of 0.9831, which proves its effectiveness in accurately identifying true negatives.

In conclusion, the integration of dilated convolution into the model architecture significantly enhances model’s performance in segmenting fundus vessels. Both qualitative and quantitative analysis indicate that choosing an appropriate dilation rate is crucial. Considering that too large a dilation rate will lead to a gridding effect in the model, which results in the loss of local feature information, the final dilation rate used in this model is chosen to be 3. This configuration balances maintaining vessel continuity and minimizing segmentation errors.

### 4.4 Discussion on ablation experiments for each module of the model

To verify the effectiveness of DS2TUNet, we performed ablation experiments on each module of the model. M1 denotes the TransUNet model, also known as the base model in this paper. M2 represents the base model combined with the double skip connection module with a dilation rate of 3. M3 signifies the base model combined with the deep supervision module. M4 denotes the DS2TUNet model, which combines both the double skip connection module with a dilation rate of 3 and the deep supervision module. The datasets used for the ablation experiments are DRIVE and CSC. The CSC is utilized for ablation experiments involving two sets of model weights: one set trained on the DRIVE as W1 and the other trained on the CHASE_DB1 as W2.

The reason for performing ablation experiments on the clinical dataset CSC is that clinical data of CSC patients contain specific imaging features and clinical manifestations, such as subretinal fluid accumulation, which may be underrepresented in publicly available datasets. Performing ablation experiments on these data allows for a better understanding of the model’s dependence on disease-specific features and ensures that it provides reliable diagnostic support in real-world clinical applications.

#### 4.4.1 Qualitative analysis

**FIGURE 10:**
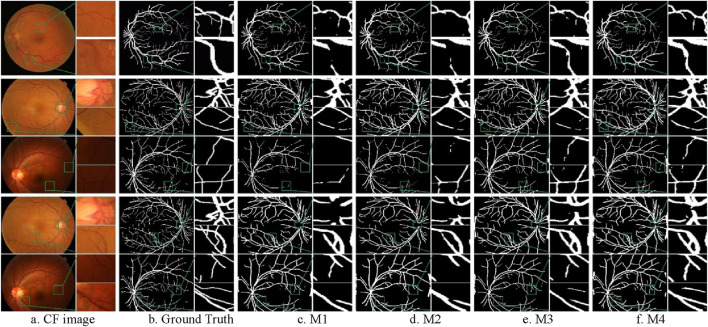
Segmentation results of ablation experiments on the CF datasets. **(A)** CF image **(B)** Ground Truth **(C)** M1 **(D)** M2 **(E)** M3 **(F)** M4.

The general trend of all models during the training period was experimentally found to be similar to the previous subsection, so the training curves is not shown at this stage. Figure 10 shows the segmentation results after adding different modules. The first row represents the segmentation results of DRIVE. The second and third rows represent the segmentation results of CSC clinical images using W1 weights. The fourth and fifth rows represent the segmentation results of the same CSC clinical images using W2 weights. The purpose of the third and fifth rows is to highlight that the model still performs well on clinical data with blood issues, such as uneven contrast, inappropriate exposure, and severe lesions.

From the Figure 10, it can be observed that the M4 model can completely segment the coarse blood vessel structure and also capture local details to segment the microvessels. The M3 model is more capable of segmenting the coarse blood vessels but tends to ignore some details. The M2 model can segment the microvessels well but is slightly less capable of segmenting the thick blood vessels. It can also be noticed that all models using W2 weights are able to segment coarser vessels but lose a lot of fine vessel information compared to models using W1. This is due to the fact that the CHASE_DB1 dataset itself is highlighting coarse vessel information.

#### 4.4.2 Quantitative analysis

The experimental results on the DRIVE and CSC datasets are presented in Tables 3 and 4 below. From the data in Table 3, it is evident that the inclusion of the double skip connection module and the deep supervision module significantly elevates the performance of the model, especially in terms of F1-score and SE. The M2 model with the introduction of the double skip connection module has been analyzed in detail in the previous experimental section. The M3 model, with the deep supervision module, performs better than the base model. This indicates that the deep supervision module ameliorates the model’s learning ability and stability. It efficiently conveys gradient information during training, which boosts the model’s capability to detect fine structures like blood vessels. The final DS2TUNet model shows a more comprehensive augmentation, with F1-score, ACC, and SE improving by 2.16%, 0.29%, and 3.7%, respectively, while SP decreases by only 0.22%.

**TABLE 3:** Results of ablation experiments on the DRIVE dataset.

Model	F1-score	ACC	SE	SP
M1	0.7979	0.9635	0.7701	**0.9845**
M2	0.8117	0.9655	0.7986	0.9831
M3	0.8005	0.9650	0.7894	0.9836
M4	**0.8195**	**0.9664**	**0.8071**	0.9823

**TABLE 4:** Results of ablation experiments on the CSC dataset.

Model	F1-score		ACC		SE		SP	
	W1	W2	W1	W2	W1	W2	W1	W2
M1	0.7607	0.7640	0.9375	0.9323	0.7943	0.7675	0.9783	0.9599
M2	0.7649	0.7730	0.9408	0.9362	0.7824	0.7602	**0.9840**	0.9659
M3	0.7722	0.7676	0.9406	0.9355	0.8059	0.7436	0.9799	0.9679
M4	**0.7747**	**0.7757**	**0.9415**	**0.9688**	**0.8072**	**0.8141**	0.9807	**0.9801**

The results of the ablation experiments of the clinical dataset CSC under both sets of weights in Table 4 also further confirm the validity of the model modules as well as the overall stability. Under W1 weights, the M4 model gets optimal results in F1-score, ACC and SE. The performance of each improved model in SP also highlights the effectiveness of each module. With W2 weights, the M4 model achieves the best results in all evaluation metrics.

The ablation experiments indicate that although the addition of individual module provides advancement over the base model, the combination of double skip connections and deep supervision delivers the most significant enhancement across all metrics. This synergy likely stems from the complementary strengths of the modules, where double skip connections facilitate better feature propagation, and deep supervision ensures more accurate gradient flow during training.

In summary, the integration of skip connections and deep supervision into the base model substantially promotes the model’s performance of vessel segmentation in clinical dataset. The results highlight the crucial role of modular developments in advancing deep learning models for medical image analysis.

### 4.5 Discussion of segmentation performance of different models

In the previous sections, we discussed the effectiveness of various modules integrated into the DS2TUNet model, particularly the double skip connections and deep supervision. We have shown through both qualitative and quantitative analyses that these modular developments significantly enhance the model’s performance in segmenting blood vessels from fundus images.

#### 4.5.1 Qualitative analysis

**FIGURE 11:**
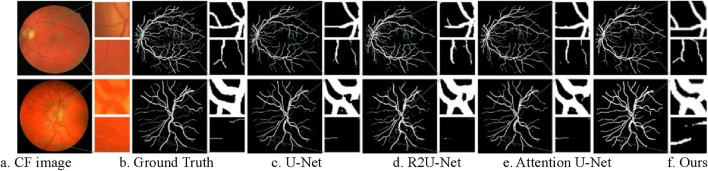
Segmentation results of different models on CF image datasets. **(A)** CF image **(B)** Ground Truth **(C)** U-Net **(D)** R2U-Net **(E)** Attention U-Net **(F)** Ours.

To intuitively compare the segmentation performance of different models, we present the visualization results of several models, including U-Net (Ronneberger et al., 2015), R2UNet (Alom et al., 2019), Attention U-Net (Schlemper et al., 2019), and the proposed method, in Figure 11. The top row represents the segmentation results of DRIVE. The bottom row represents the segmentation results of CHASE_DB1.

As the original model, U-Net produces unsatisfactory segmentation results. Many thin vessels are missing in the segmentation masks generated by U-Net, particularly in CHASE_DB1 dataset. Additionally, the localization of thick vessels is often inaccurate, as seen in the DRIVE dataset, with numerous misclassifications. R2U-Net, which utilizes a recurrent convolutional structure to accumulate features, captures more detailed information, but this detail tends to be fragmented, resulting in poor continuity, especially for thin vessels on CHASE_DB1. Attention U-Net improves on this by embedding a gated attention mechanism that enhances vessel information propagation while suppressing irrelevant features, yielding better segmentation outcomes than U-Net and R2U-Net.

The proposed method further improves segmentation for both thick and thin vessels over Attention U-Net. When comparing thin vessel segmentation across all three datasets, the proposed method captures more detailed thin vessel features with improved continuity. For thick vessels, it achieves more accurate localization and segmentation. Overall, the proposed method demonstrates greater robustness and consistently produces segmentation results that are closest to the ground truth across all three datasets.

#### 4.5.2 Quantitative analysis

To evaluate the effectiveness of the DS2TUNet model, we perform a quantitative analysis with several established segmentation models using the DRIVE and CHASE_DB1 datasets. Tables 5 and 6 present the detailed evaluation metrics of the DS2TUNet model against its counterparts. The results exhibit how DS2TUNet stands in terms of its ability to accurately and reliably segment blood vessels compared to other models. This comparative analysis helps to understand the practical benefits and potential areas for improvement in DS2TUNet’s performance, offering a comprehensive view of its effectiveness in real-world applications.

**TABLE 5:** Comparison of different models in DRIVE dataset.

Model	F1-score	ACC	SE	SP
Ronneberger et al. (2015)	0.8142	0.9531	0.7537	0.9820
Fu et al. (2016)	---	0.9533	0.7603	0.9776
Yan et al. (2018a)	---	0.9542	0.7653	0.9818
Alom et al. (2019)	0.8171	0.9556	0.7792	0.9813
Li et al. (2020)	0.8192	0.9557	0.7890	0.9799
Feng et al. (2020)	---	0.9528	0.7625	0.9809
Lv et al. (2020)	0.8216	0.9558	0.7941	0.9798
Du et al. (2021)	0.8204	0.9556	0.7814	0.9810
Li et al. (2016)	---	0.9527	0.7569	0.9816
Schlemper et al. (2019)	0.8218	0.9610	0.7819	**0.9834**
Alvarado-Carrillo and Dalmau-Cedeño, (2022)	0.8269	0.9575	0.7966	0.9810
Liu et al. (2023)	0.8229	0.9561	0.7985	0.9791
Guo, (2023)	**0.8289**	0.9571	0.7934	0.9810
Kumar et al. (2023)	0.8262	0.9568	0.8053	0.9789
Ours	0.8195	**0.9664**	**0.8071**	0.9823

**TABLE 6:** Comparison of different models in CHASE_DB1 dataset.

Model	F1-score	ACC	SE	SP
Ronneberger et al. (2015)	0.7936	0.9604	0.7621	0.9824
Orlando et al. (2016)	0.7332	---	0.7277	0.9712
Yan et al. (2018a)	---	0.9610	0.7633	0.9809
Alom et al. (2019)	0.7810	0.9622	0.7459	0.9836
Li et al. (2020)	0.8037	0.9620	0.7798	0.9822
Lv et al. (2020)	0.7892	0.9608	0.8176	0.9704
Du et al. (2021)	0.7813	0.9590	0.8195	0.9727
Li et al. (2016)	---	0.9581	0.7507	0.9793
Schlemper et al. (2019)	0.8180	0.9680	0.7954	0.9841
Alvarado-Carrillo and Dalmau-Cedeño, (2022)	0.8098	0.9653	0.8042	0.9826
Liu et al. (2023)	0.8236	0.9672	0.8020	0.9794
Guo, (2023)	0.8021	0.9650	0.7645	0.9846
Kumar et al. (2023)	0.7875	0.9591	**0.8443**	0.9704
Ours	**0.8362**	**0.9741**	0.8101	**0.9869**

By comparing different models on the DRIVE dataset, our model certificates significant performance across several key metrics. Relative to the listed methods, our model accomplishes the highest ACC value of 0.9664, approximately 0.54% better than the top-ranked model. Our model excels in SE, reaching 0.8071, about 0.18% higher than the other model, showcasing its strong capability to correctly identify blood vessels. Additionally, our model also performs well in the SP, achieving a result of 0.9823 slightly lower than Schlemper’s result. Other models also show impressive performance. For example, Guo’s model attains an optimal F1-score of 0.8289. Although our model’s F1-score of 0.8195 is slightly lower than some other models, it still maintains a considerable competitive edge. The above data highlights the effectiveness and reliability of our model in the task of vessel segmentation on the DRIVE dataset.

Similarly, based on the comparison of different models on the CHASE_DB1 dataset, our model exhibits notable performance across various metrics. It reaches the highest F1-score of 0.8362, outperforming other models listed with an improvement of roughly 1.26% over the next best model. In terms of ACC, our model also excels with a value of 0.9741, significantly higher than other models, with an increment of about 0.61% over the closest model. The SE of our model is 0.8101, which is competitive, though slightly lower than Kumar’s result, which reports the highest SE of 0.8443. This difference of nearly 3.42% is offset by our model’s superior performance in other metrics. Additionally, our model achieves the highest SP, i.e. 0.9869. These results underscore our model’s effectiveness and reliability in vessel segmentation tasks on the CHASE_DB1 dataset.

In summary, our model exhibited notable robustness and reliability in vessel segmentation tasks, attaining strong F1-score, ACC, and leading SE and SP across both datasets. These combined results highlight the model’s capability to perform precise fundus vessel segmentation, making it a valuable tool for fundus image analysis.

### 4.6 Segmentation effects on the OCTA dataset

OCTA images are acquired using optical coherence tomography, which mainly depict the structure of the vessel network with high resolution and obvious vessel contrast and are suitable for detailed analysis of the microstructure of the retinal vasculature and its lesions. CF images, on the other hand, are acquired by conventional fundus photography, and their vessel details are not as clear as those of OCTA, which is only suitable for routine fundus examination. Therefore, it is necessary to segment the blood vessels in OCTA images. The experiments performed above are based on the CF datasets. To further investigate the generalization ability of the model, we conduct experiments on the OCTA dataset ROSE-1. The experiments include ablation experiments of different modules in the model and performance comparison experiments with different models.

#### 4.6.1 Ablation experiments

**FIGURE 12:**
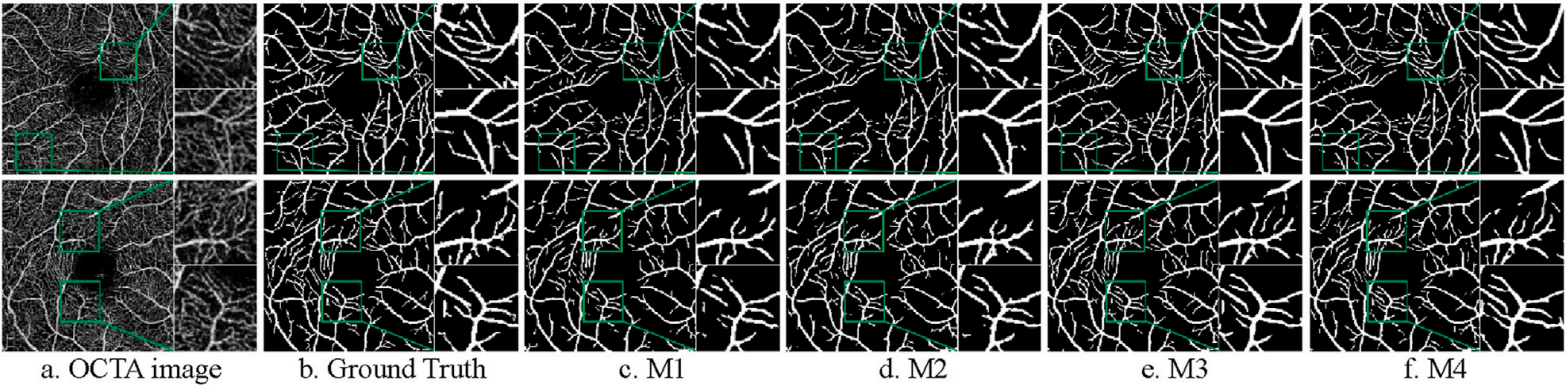
Segmentation results of ablation experiments on the ROSE-1 dataset. **(A)** OCTA image **(B)** Ground Truth **(C)** M1 **(D)** M2 **(E)** M3 **(F)** M4.

The model nomenclature for this part of the ablation experiments is the same as that of the ablation experiments on the CF dataset. The segmentation results are shown in Figure 12. The result of the quantitative analysis is shown in Table 7. By comparing with the ground truth, we observe that the segmentation results of models M2 and M3 show advancements after integrating different modules, which alleviates issues of vessel omission and over-segmentation seen in the initial model. Additionally, it is evident that the final model M4 yields results most closely aligned with the ground truth.

**TABLE 7:** Results of ablation experiments on the ROSE-1 dataset.

Model	F1-score	ACC	SE	SP
M1	0.8323	0.9559	0.7947	**0.9817**
M2	0.8345	**0.9563**	0.8043	0.9805
M3	0.8390	0.9548	0.8553	0.9709
M4	**0.8425**	0.9557	**0.8586**	0.9713

As shown in Table 7, except for the SP value where the basic model performs best, the improved models outperform in all other indicators. The data of M2 and M3 can reflect that the performance of the model segmentation OCTA dataset is elevated after adding the double skip connection module and the deep supervision module. The M2 model performed well in ACC, with a maximum of 0.9563 among all models. The M3 model is 6.06% higher than the basic model M1 in terms of SE. The final model M4 achieved the maximum value on F1-score and SE. Through ablation experiments, we further illustrate the effectiveness of each module and the effectiveness of the overall model for vessel segmentation of the OCTA dataset from a quantitative perspective.

#### 4.6.2 Performance comparison experiments

**FIGURE 13:**
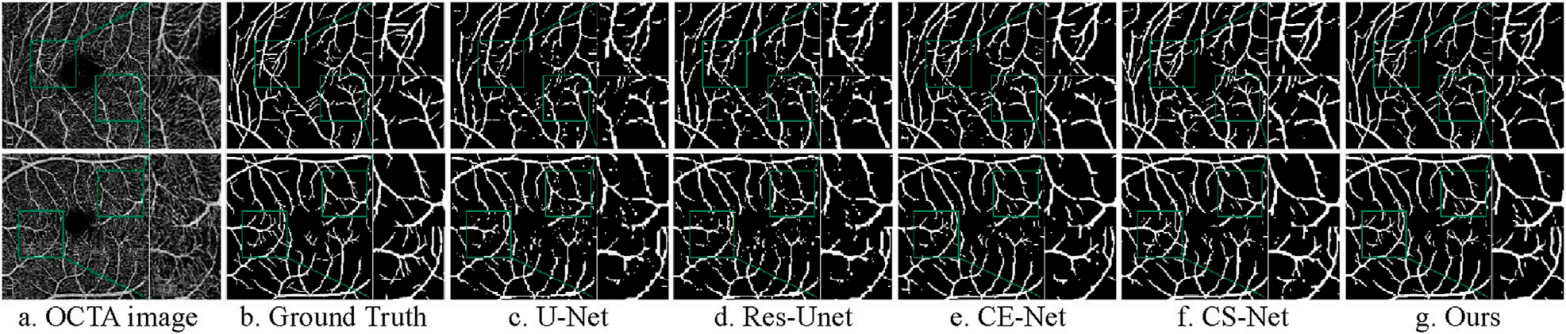
Segmentation results of different models on ROSE-1 dataset. **(A)** OCTA image **(B)** Ground Truth **(C)** U-Net **(D)** Res-Unet **(E)** CE-Net **(F)** CS-Net **(G)** Ours.

To evaluate the ability of the proposed DS2TUNet to segment fundus vessels in OCTA images, this section compares the proposed method with four advanced methods on the ROSE-1 dataset, including U-Net (Ronneberger et al., 2015), Res-Unet (Zhang et al., 2018), CE-Net (Gu et al., 2019), and CS-Net (Mou et al., 2019). Res-Unet enhances U-Net by adding a weighted attention mechanism and modifying the skip connections to improve fundus vessel segmentation in color fundus images. The segmentation results of fundus vessels are shown in Figure 13. The quantitative results of the experiments are as follows Table 8.

**TABLE 8:** Comparison of different models in ROSE-1 dataset.

Model	F1-score	ACC	SE	SP
Ronneberger et al. (2015)	0.7116	0.8955	0.8125	**0.9783**
Zhang et al. (2018)	0.7461	0.9098	**0.9170**	0.9578
Gu et al. (2019)	0.7511	0.9121	0.9167	0.9605
Jin et al. (2019b)	0.7505	0.9118	---	---
Mou et al. (2019)	0.7608	0.9152	0.8641	0.9754
Yan et al. (2018b)	0.7663	0.9179	---	---
Ma et al. (2020)	0.7697	0.9182	---	---
Ours	**0.8425**	**0.9557**	0.8586	0.9713

Upon observation, the first three comparison methods produce more white specks, indicating a higher occurrence of vessel disconnection and poor continuity in the segmented fundus vessels. Compared to the first three methods, CS-Net achieves better vessel continuity, likely due to its attention to the elongated tubular structure of vessels and its design incorporating this prior knowledge. CS-Net employs 1 × 3 and 3 × 1 kernel convolutions in the attention mechanism to capture vessel junctions. By comparing the magnified regions within the green boxes, the proposed method results in fewer vessel disconnections and produces clearer vessel terminations.

According to the comparative analysis of various models on the ROSE-1 dataset, our model demonstrates superior performance across critical metrics. It achieves the highest F1-score of 0.8425, outperforming all other models by approximately 7.28%. Additionally, our model achieves an ACC of 0.9557. This score is about 3.75% higher than that of the best listed model. In terms of SE and SP, our model performs slightly lower compared to some of the other models in the table, which indicates that the model still has room for improvement. These metrics highlight the model’s robust capacity for precise blood vessel segmentation and underscore its reliability and effectiveness in vessel segmentation tasks on the ROSE-1 dataset.

In conclusion, the DS2TUNet, model not only performs well in the task of fundus vessel segmentation on the CF, datasets, but also shows strong performance on the OCTA, dataset. This highlights the generalization ability of the model, making it an important tool for multimodal retinal image segmentation.

## 5 Conclusion and future work

In this study, we put forward DS2TUNet, a novel deep learning model for fundus vessel segmentation that integrates the dual downsampling operation, the double skip connection module, and the deep supervision module. Our approach leverages the advantages of dilated convolutions, ResNetV2, and Transformer to augment feature extraction and improve segmentation accuracy.

By incorporating dilated convolutions, DS2TUNet effectively captures both local and global features, addressing the complexity of retinal vessel structures. The dual downsampling operation, which combines standard and dilated convolutions, significantly boosts contextual information without increasing computational complexity. The double skip connection module improves feature fusion and preserves detailed information across different resolution levels. It mitigates the loss of contextual information during downsampling, resulting in more precise segmentation results. The deep supervision module ensures efficient gradient flow during training, which reduces the risk of gradient vanishing and enhances model stability. This module contributes to the accurate detection of both coarse and fine vessel structures. DS2TUNet outperforms several state-of-the-art models on the DRIVE and CHASE_DB1 datasets, achieving the highest ACC and SP, which demonstrates its robustness and reliability in fundus vessel segmentation tasks. Additionally, the model shows strong generalization ability when tested on OCTA dataset, highlighting its applicability to various types of retinal images and its utility in clinical settings.

The proposed DS2TUNet model addresses key challenges in fundus vessel segmentation by effectively balancing the extraction of local and global features and preserving detailed information through its innovative network architecture. This study contributes to the advancement of retinal image analysis, providing a reliable tool for diagnosing and monitoring various ocular diseases. Future research can explore the following directions: enhancing the training dataset with more diverse and augmented images to improve model generalization and robustness and integrating DS2TUNet into real-world clinical environments to validate its practical utility and performance.

## Data Availability

The raw data supporting the conclusions of this article will be made available by the authors, without undue reservation.
